# A Fully Transparent Flexible Sensor for Cryogenic Temperatures Based on High Strength Metallurgical Graphene

**DOI:** 10.3390/s17010051

**Published:** 2016-12-28

**Authors:** Ryszard Pawlak, Marcin Lebioda, Jacek Rymaszewski, Witold Szymanski, Lukasz Kolodziejczyk, Piotr Kula

**Affiliations:** 1 Institute of Electrical Engineering Systems, Lodz University of Technology, 90-924 Lodz, Poland; marcin.lebioda@p.lodz.pl (M.L.); jacek.rymaszewski@p.lodz.pl (J.R.); 2 Institute of Materials Science and Engineering, Lodz University of Technology, 90-924 Lodz, Poland; witold.szymanski@p.lodz.pl (W.S.); lukasz.kolodziejczyk@p.lodz.pl (L.K.); piotr.kula@p.lodz.pl (P.K.)

**Keywords:** low-temperature, cryogenic, sensors, graphene

## Abstract

Low-temperature electronics operating in below zero temperatures or even below the lower limit of the common −65 to 125 °C temperature range are essential in medical diagnostics, in space exploration and aviation, in processing and storage of food and mainly in scientific research, like superconducting materials engineering and their applications—superconducting magnets, superconducting energy storage, and magnetic levitation systems. Such electronic devices demand special approach to the materials used in passive elements and sensors. The main goal of this work was the implementation of a fully transparent, flexible cryogenic temperature sensor with graphene structures as sensing element. Electrodes were made of transparent ITO (Indium Tin Oxide) or ITO/Ag/ITO conductive layers by laser ablation and finally encapsulated in a polymer coating. A helium closed-cycle cryostat has been used in measurements of the electrical properties of these graphene-based temperature sensors under cryogenic conditions. The sensors were repeatedly cooled from room temperature to cryogenic temperature. Graphene structures were characterized using Raman spectroscopy. The observation of the resistance changes as a function of temperature indicates the potential use of graphene layers in the construction of temperature sensors. The temperature characteristics of the analyzed graphene sensors exhibit no clear anomalies or strong non-linearity in the entire studied temperature range (as compared to the typical carbon sensor).

## 1. Introduction

The application of transparent conductive films and multilayer films for resistance temperature detectors in cryogenic systems was discussed for the first time in [1]. Graphene is one of the most promising materials for a diversity of modern technological applications due to its excellent electrical, optical, thermal, mechanical, electrochemical and structural characteristics [2,3,4]. The exceptionally high electrical conductivity of graphene combined with its transparency, flexibility and mechanical strength, make it suitable for microelectronic devices (Field Effect Transistors—FETs), photonics and optoelectronic systems, passive electronic elements and for sensing applications [3,5,6,7]. We propose for the first time a sensor with a graphene layer as sensitive element designed for cryogenic temperature measurement. The electrodes of the sensor are shaped using transparent conductor film on a polymer substrate using laser direct patterning, so the whole device is transparent and flexible. Encapsulation with a polymer transparent cover protects the sensor against any damage. The as-fabricated sensors have a low thermal capacity due to their construction. Changes in resistance of the sensors using HSMG^®^ (High Strength Metallurgical Graphene) graphene layers are measurable and significant. The sensors’ sensitivity in the range of cryogenic temperatures (particularly below 50 K) is much higher than the sensitivity of comparable resistive (metallic) temperature sensors (RTD). Temperature sensors made of the graphene film transferred on the polymeric substrate have significantly better flexibility and resistance to thermal stresses in comparison to bulk graphite sensors. Application of thin conductive polymeric films (ITO—Indium Tin Oxide and AgHT^TM^) allowed us to obtain the sensors with electrodes characterized by high optical transparency and therefore may be applicable in low-temperature optical studies (UV—Ultraviolet).

The crucial features for flexible, transparent temperature sensors are their electrical conductivity, thermal properties, and the optical transmittance of the materials used. These properties of graphene and transparent conducting oxides films are briefly discussed below.

The electrical conductivity of large-surface graphene sheets is a very complex issue, resulting from the characteristics of their carrier transport. The electronic properties of graphene are mostly considered and investigated from the viewpoint of its use as a material that could replace silicon in microelectronic devices controlled by electric fields (MOSFETs). The experimentally measured minimum conductivity in graphene (б_min_ ≈ 4 e^2^/h; resistivity ρ ≈ 6.5 kΩ/sq) is close to the theoretically predicted quantum conductivity for Dirac fermions [2]. A rapid decrease in resistivity occurs after gate voltage switching due to the increasing carrier density. The resistivity measurements at 5 K revealed near-ballistic transport in samples of dimensions about 2 μm [8]. In longer graphene samples carrier transport has a diffusive character due to elastic and inelastic scattering [6,9]. There are different sources of elastic scattering in graphene: charged impurities, defects, absorbed atoms and those factors connected with the structural morphology of graphene sheets such as ripples and roughness on the graphene surface. Monolayer graphene in fact is not a perfectly flat 2D structure but rather, especially when placed on a substrate, it will adjust to the underlying surface roughness. The ripples of graphene can create a long-range scattering potential leading to important charge-carrier limitations (rises of resistivity) [10,11]. Discussion of the inelastic scattering from the phonons could be limited practically to the longitudinal acoustic phonons. The contribution of phonon scattering to total resistivity (on the order of some kΩ) of graphene samples is of the order of 100 Ω at 300 K [12,13]. In graphene samples of larger dimensions, e.g., in graphene nanoribbons with small widths of some tens of nanometers and lengths of some tens of micrometers, lateral confinement of the charge carriers causes the creation of an energy gap tuned by the appropriate choice of ribbon width [14,15]. There are also other causes of charge scattering, such as folding of the graphene sheets, phonons in the graphene or interfacial phonons between the graphene and the supporting substrate. Large-area graphene sheets are a 2D polycrystalline material consisting of domain and grains [7]. The grain boundary, depending of its structure, can manifest whole reflection or high transparency toward charge carrier transport [16]. In devices using as functional element large area graphene sheetd (e.g., sensors) contact/graphene interface phenomena should be taken into account. An additional charge inhomogeneity occurs in the vicinity of the contact but could extend hundreds of nanometers from the contact. Studies have proven that the contact resistance (Ti/Au-graphene) includes a component independent of the gate voltage of a value of about 800 Ω∙µm, which is insensitive to temperature changes [17]. The contact resistance (700 ± 500 Ω∙µm) was found to be independent of the metal work function (for Ti, Ag, Co, Cr, Fe, Ni, Pd) [18].

The thermal conductivity *κ* of graphene is extremely high and exceeds 5000 W/mK at room temperature [19]. These outstanding thermal properties seem to be very attractive for microelectronic and sensor applications. The predominant contribution to the high thermal conductivity of single layer graphene corresponds to acoustic phonons with a mean free path of 500–1000 nm, while the contribution of electrons is negligible [20,21,22]. Because of the long phonons’ mean free path the thermal properties are dependent on the sample size and grain size and orientation. Defects and impurities as well as stresses in the graphene structure reduce the thermal conductivity [23,24]. The thermal properties of graphene are strongly affected by the influence of the supporting material [21]. The thermal conductivity of about 600 W/mK for single layer graphene supported on a SiO_2_ membrane proved to be much less than that of suspended graphene (3000–5000 W/mK, although it should be noted that the *κ* value of supported graphene was still greater than that of metals (Cu, Ag—*κ* > 400 W/mK). The phonons’ mean free path in supported graphene, predicted theoretically and estimated from measurements, is about 100 nm [21]. The interactions between graphene layers and substrate surfaces could be different for the various metals and dielectrics used as substrate materials and the method of graphene synthesis, so different scales of reduction of thermal conductivity could be observed [25]. 

An optical transmittance of single layer graphene equal to 97.7% was theoretically derived, assuming a constant high-frequency conductivity for Dirac fermions through the visible range of the spectrum, and proved experimentally [5,26]. This transparency is quite constant for wavelengths from 300 to 2500 nm, with a small decrease at about 270 nm [3,27]. The optical transparency for few layer graphene is reduced about 2.3% for every layer. 

The above brief considerations suggest that, especially in applications that use graphene sheets of large size (of the order of millimeters), the dependence of the electrical, thermal and optical properties on methods for graphene synthesis, sample size, grain structure, purity, homogeneity and substrate type must be taken into account.

Although graphene has attracted greatest interest as a material for active microelectronic devices (graphene FETs), its exceptionally high electrical conductivity and furthermore its transparency, flexibility and mechanical strength, make it also suitable for passive electronic elements. Recently the requirements of modern optoelectronic technologies, such as photovoltaic technology, flat panel displays, OLEDs (Organic Light-Emitting Diodes) and optoelectronic devices, have caused the rapid development of new conductive transparent materials in the form of thin layers [28,29,30,31,32]. Of great importance for practical applications are conductive oxides transparent layers, usually indium tin oxide (ITO). The increasing price of indium and the lack of stretchability of ITO has inspired research for replacing ITO with other transparent conductive materials, such as ZnO, carbon nanotubes (CNTs) [33], conducting polymers like poly(3,4-ethylenedioxythiophene) polystyrene sulfonate—PEDOT:PSS [34], thin transparent metal films [35] and multilayer systems that consist of two outer layers of oxides (ZnO, ITO) and a thin metallic film (Ag, Cu, Au) between them, e.g., ITO/Ag/ITO multilayers. The main purpose of many of these studies was producing these multilayers on transparent electrodes for organic solar cells or OLEDs [36,37], transparent UWB (Ultra-WideBand) antennas [38,39] and also as EMI (ElectroMagnetic Interference) shielding materials [40].

Recently, several innovative temperature sensors based on graphene have been developed, thus providing an alternative to conventional rigid ceramics. Mono- or bi-layer graphene nanofabricated on a silicon substrate has been used as a thermally sensitive element, however no flexibility has been achieved in such a system. Al-Mumen et al. [41] studied the temperature sensing behavior of mono-, bi- and few layer graphene exfoliated from graphite. The resistance temperature coefficient (TCR) was determined, which was about −0.007 K^−1^ for the bi-layer graphene, about −0.003 K^−1^ for the monolayer graphene, and about −0.0015 K^−1^ for the few-layer graphene, respectively. The bilayer graphene had the highest negative TCR, measured as the temperature changed between RT and 80 °C. Kong et al. [42] fabricated a mechanically stable graphene electrode by its direct micropatterning onto a flexible polymer. The negative temperature coefficient (NTC) of the graphene electrode was similar to that of conventional NTC materials, however its response time was faster by an order of magnitude. Trung et al. [43] proposed a flexible and very sensitive sensor based on reduced graphene oxide transferred onto a transparent polymer substrate. Yang et al. [44] invented a wearable sensor by incorporation of graphene nanowalls into PDMS which value of resistance temperature coefficient exceeded by threefold the values typical for conventional sensors. Yan et al. [45] demonstrated stretchable graphene thermistors with intrinsic high stretchability that were fabricated through a lithographic filtration method based on conductive AgNW electrodes and a resistive graphene detection channel. The devices were stretched up to 50% and could maintain their functionality even in highly stretched states. Bendi et al. [46] reported a self-powered thermistor which utilizes the formation of p-i-n junctions on a graphene monolayer using ferroelectric polymer PVDF-TrFE (poly[(vinylidenefluoride-co-trifluoroethylene)]) that generates current changes when subjected to thermal stimulation (40–110 °C) under no external perturbation.

These excellent properties indicate graphene is a candidate for the preparation of the transparent, flexible electrodes, e.g., instead of ITO [47]. Many interesting nanodevices and structures using electrodes of graphene have been proposed, as organic FETs [47], flexible transparent piezoelectric energy harvesters [48] or transparent resistive memory [49]. Double layers consisting of graphene sheets and ITO layers were applied as source-drain electrodes in a thin film transistor (InGaZnO) [50] and a n GaN light emitting diode [51]. Quite recently a multilayer structure consisting of a graphene layer formed on Cu foil and doped with Au nanoparticles (anode) and with Ag-nanowires (cathode) were used to creating a through transparent quantum dot light-emitting diode [52]. Inks based on graphene obtained from the reduction of graphene oxide (GO) and suspended in liquids enabled fabrication of flexible sensors, for example, a transparent acoustic actuator [53] and a sensor for the simultaneous measurement of pressure and temperature [54]. In the construction of the organic electrochemical transistors electrodes were doped with graphene flakes prepared by ultrasonic exfoliation from graphite [55].

## 2. Materials and Methods

### 2.1. Formation of Transparent Electrodes by Laser Ablation

To create electrodes for cryogenic temperature sensors two different conductive films were used, namely commonly used ITO and ITO/Ag/ITO film (AgHT^™^) with surface resistance of 15 Ω/sq and 4 Ω/sq, respectively. AgHT™ is a highly conductive film on a polyester substrate which is significant in shielding against EMI/RFI (ElectroMagnetic Interference/ Radio-Frequency Interference) and also infrared heat rejection. Its good electrical conductivity, high optical transparency and flexibility determine applications of this material in membrane switches, photovoltaic structures, displays, and passive elements of flexible and transparent electronics. The ITO film on PEN (Poly(ethylene 2,6-naphthalate) substrate had a thickness of 125 nm and an ITO/Ag/ITO film thickness of 150 nm.

Laser Direct Writing (LDW) methods for nanometer films are used in the manufacture of flexible electronic circuits and sensors on the sub-millimeter scale. It is well known that laser processing of thin layers of nanometer thickness can be performed using laser beams of short wavelength and short pulse duration. Thin functional layers (ITO, AgHT, carbon nanotube layers) on transparent substrate materials (polyester, PET) have similar ablation threshold fluences, therefore damage of the substrate layers during laser processing should be avoided. Processing of most materials demands applying of UV laser beams with femto- or picosecond pulses to ensure the best effects. Patterning of ITO thin films was performed using laser beam pulses of ultraviolet to infrared wavelength of picosecond, femtosecond or nanosecond duration [56,57,58,59,60]. Laser direct writing was applied to electrode patterning for flat panel displays [61], fabricating a miniature transparent gas flow meter [62], electrode isolation in ITO layer on substrates used in the mobile phones [63], a pentacene thin film transistor (TFT) with source and drain electrodes patterned in ITO [64], and producing matrix array of OLEDs [65]. 

**Figure 1:**
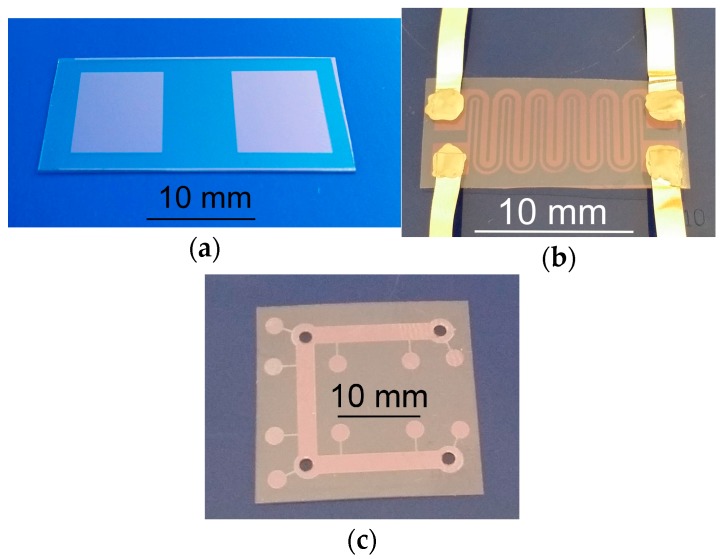
Examples of structures prepared by laser ablation: (**a**) electrodes for cryogenic sensors made in ITO layer on PEN substrate; (**b**) micro-heater in ITO/Ag/ITO layer with silver leads; (**c**) test structure for examination of electrical properties of ITO on PEN.

The main goal of our former research was to establish the possibility of using a single mode fiber laser in micromachining considering the quality of the obtained structures and their smallest dimensions while maintaining acceptable quality [66]. We have shown that the LDW method by nanosecond laser ablation ensures good conditions for prototyping structures with very high pattern fidelity. Among other uses contacts for prototyping of OLED structures [66] and samples of two types of conductive path have been prepared. The first had a meandering shape and was used in resistivity measurements and the second had the shape of a cross and was used in Hall effect measurements (Figure 1) [1].

The optical transparency was not changed, although some thermal effects were observed after laser treatment. In case of the ITO/Ag/ITO layer even improvement of the optical transmission after laser micromachining was noted. Previous studies have shown that structures patterned in AgHT conductive film can be useful for sensors in cryogenic systems and passive elements in flexible electronics [1]. A redENERGY G3 SM 20W single mode fiber laser (SPI Lasers UK Ltd., Southampton, UK), which guaranties high quality of the beam (M2 < 1.3) was used to manufacture the electrodes for cryogenic temperature sensor with graphene sensitive layer. The laser beam was scanned by a 2-Axis Scan Head (Xtreme, Nutfield Technology. Inc., Hudson, NH, USA) equipped with a 100 mm F-theta lens and was controlled by the SB-1P Waverunner software (Xtreme, Nutfield Technology. Inc., Hudson, NH, USA). The optimal parameters for creating electrodes were as follows: pulse energy—120 µJ; pulse duration—25 ns; pulse repetition frequency—72 kHz; scanning velocity—800 mm/s and for ITO film: pulse energy—145 µJ; pulse duration—25 ns; pulse repetition frequency—80 kHz; scanning velocity—1500 mm/s.

**Figure 2:**
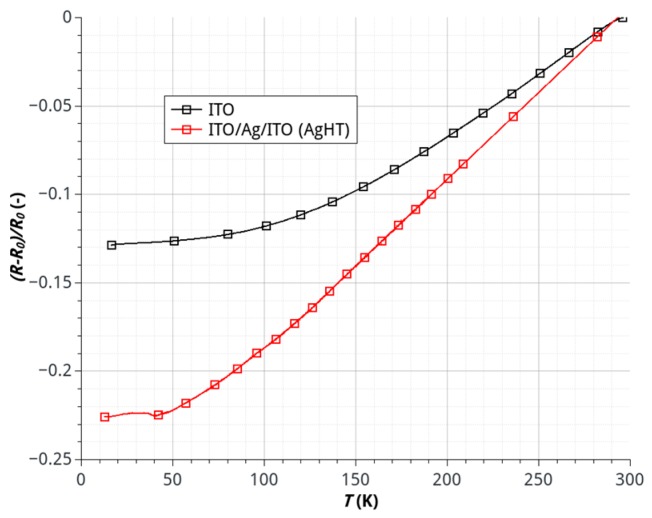
Temperature dependence of resistance of electrodes prepared from conductive polymers.

The characteristics electrode resistance changes with temperature in the range of 12–300 K are shown in Figure 2. Studies have proven that changes of substrate resistance, due to temperature changes, are continuous and repetitive in the analyzed range. This means that the conductive layer is continuous in a wide range of temperature, and the substrate is not permanently deformed or damaged.

### 2.2. Synthesis and Transfer of Graphene Film

The high strength metallurgical graphene (HSMG^®^) sheets were synthesized in an industrially scaled thermochemical facility based on the process described in previous papers [67,68,69]. The copper/nickel composite substrate was heated in an argon atmosphere at a pressure of 100 kPa. After this step the chamber was evacuated to a pressure of 2 Pa and a mixture of acetylene, hydrogen and ethylene (ratio 2:1:2), at a partial pressure of 3 kPa, has been simultaneously introduced with argon for 1 min. Finally, the substrate with graphene was cooled stepwise to RT in an argon atmosphere at a pressure of 100 kPa. 

The modified transfer procedure of HSMG^®^ graphene for temperature sensors and reference samples preparations was used. The procedure is based on the commonly used method of graphene transfer from metallic substrates onto Poly(methyl methacrylate)—PMMA foil, which is described in details in our previous paper [70]. Methods utilizing a thin film of PMMA as a graphene supporting material and their variations are the most frequently used methods for transfer of 2D materials (graphene) onto any substrate. Wrinkles and cracks were observed on the graphene after the transfer process. An extensive explanation for the formation of these defects is presented in our previous work [70].

The production method of HSMG^®^ allowed manufacturing of a single layer of graphene. The intentional and controlled modifications of the synthesis process lead to a graphene-like material (G-LM) with a slightly lower value of resistance/square. 

Analysis of the graphene transferred onto reference samples was carried out using an inVia Raman spectroscope (Renishaw, New Mills, UK). All acquisitions of Raman spectra for graphene structure were performed with using an Ar+ laser at a laser excitation wavelength λ = 532 nm, exposure time was 10 s; signal was averaged from three times repeated exposition per spot. The maximum laser output power was 29.3 mW but the test was carried out at only 10% of the output power. Raman scattering was observed for the 1200–3100 cm^−1^ wavenumber range. The acquisition settings listed above did not cause any changes on the surface, like damage by local heating. Data processing was performed using the PeakFit software (Systat Software Inc., London, UK). Gauss–Lorentz curves were used for spectra deconvolution. Data obtained from the deconvolution for example: peak positions, intensities, half-widths were used for the calculation of characteristic peak ratios of graphene structures which were used in further analysis are provided in following tables (Table 1 and Table 2).

### 2.3. Encapsulation of TCO/HSMG^®^ or G-LM Samples

**Figure 3:**
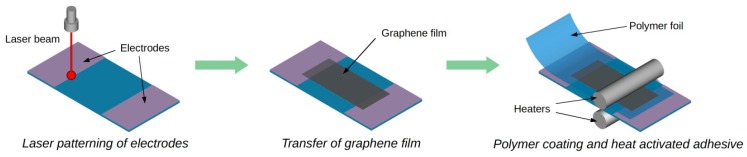
Method of manufacturing graphene cryogenic temperature sensor.

The construction of the graphene cryogenic temperature sensor should eliminate or reduce the sensitivity of the sensor to stimuli other than the temperature. Particularly important is the ability to control the influence of gases on the electrical properties of graphene. This means that the graphene should be insulated from the influence of gas (vacuum inside) or the sensor should be filled with gas in a controlled way (gas inside). Moreover, the structure should provide a simple method of implementing the electrical connections. The reduction of heat exchange with the room temperature environment and heat capacity of the cooled part are just as important in cryogenic systems. The presented transfer of graphene film on a polyester substrate with highly conductive electrodes provided for a small sensor thermal capacity and reduced the heat transfer through the electrodes. The LDW method enabled patterning of electrodes in various shapes, while maintaining the transparency of the electrodes and the substrate. The transparency of the sensor is a unique feature of the presented encapsulation technology. In addition, the small cross section of the thin film electrodes effectively reduces the heat transfer to the cryogenic. Features of the applied substrate and the method of electrode patterning favored the use of a protective thin transparent polymer foil (about 10 μm). The protective film coated on graphene and partially on the electrodes isolates these elements electrically and chemically from the environment (Figure 3). 

**Figure 4:**
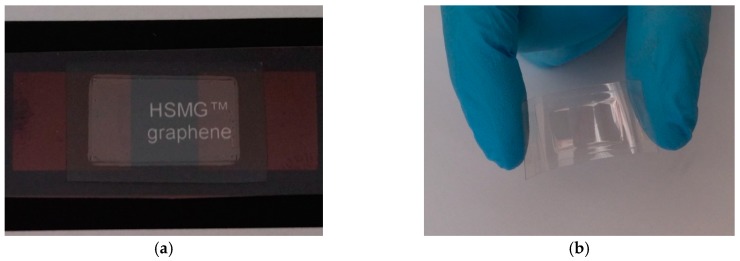
Examples of fully transparent temperature sensors: (**a**) transparency of cryogenic sensor—photo of the sensor on a black background with a white label; (**b**) stretchability of the sensor.

Integration of the polymer film onto the substrate is the result of a thermal activation adhesive. Proper preparation of the protective layer made it connect only to the substrate and does not affect the graphene film. The encapsulation process did not adversely affect the electrical and optical properties of the sensors. The encapsulation process may be performed either under vacuum or in a gas. This is important because thus can be used to change the parameters of the temperature sensor by doping the gas. The sensors retain the transparency and flexibility of conventional polymers after encapsulation process. The proposed encapsulation of the samples provides various options for electrical connections between the sensor and measurement systems. Two types of electrical joints were applied: 1st—a pressed contact as in butt joints, where a thin silver foil (35 μm) has been used and 2nd—an adhesive contact, where the electrically conductive silver-epoxy Elpox AX 15s (Amepox Microelectronics Ltd., Lodz, Poland) has been applied. No effects of the various types of contacts on sensor properties were observed. The proposed technology of encapsulation ensures the possibility of forming fully transparent sensors with different size and shapes (Figure 4). 

**Figure 5:**
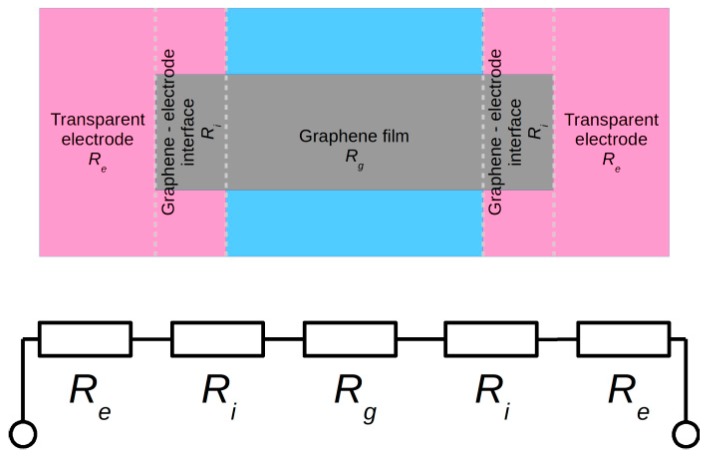
Equivalent circuit of graphene temperature sensor.

Additionally, it is possible to realize many different sensors at once using the same structure (e.g., an array temperature sensor, temperature sensor and a gas sensor). A graphene monolayer (HSMG^®^) and graphene-like material (G-LM) were used in the construction of sensors. The total resistance of the manufactured sensor is the sum of the resistance of electrodes *R_e_*, the resistance of graphene-electrode interfaces *R_i_* and the resistance of the graphene layer *R_g_* (HSMG^®^ or G-LM) (Figure 5). 

The temperature affects the all listed resistances to a different extent. Therefore, proper selection of materials for electrode construction is particularly important. In the proposed solution, the substrates with electrodes were made of ITO/Ag/ITO AgHT (4 Ω/sq) and ITO (15–100 Ω/sq). The resistance value of the graphene layer is relatively high (>20 kΩ/sq). A significant difference of the resistance of the graphene layer and the resistance of the substrate, resulting from the different thickness of graphene monolayer and electrodes (~120 nm), minimizes the participation of the electrode resistance and the interface between the graphene and electrodes in the total resistance of the sensor.

### 2.4. Instrumentation and Experimental Procedure

**Figure 6:**
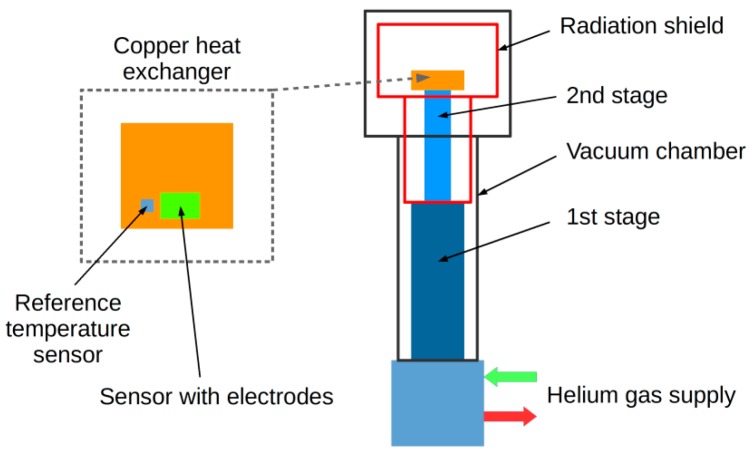
Scheme of cooling circuit.

All tests and measurements of electrical properties of graphene cryogenic temperature sensors were performed in a helium closed-cycle DE-210 cryostat (Advanced Research Systems, Inc., Macungie, PA, USA). The sensors were cyclically cooled from room temperature (295 K) to cryogenic temperature (20 K) at a rate of about 0.1 K/s. The tested graphene sensors and reference temperature probes were placed directly on the surface of the massive copper heat exchanger (a copper block 80 mm × 80 mm × 10 mm). The encapsulation of the sensor provided the electrical and chemical isolation of the sensor from the environment and other elements of the cryogenic system. In order to eliminate the temperature gradient along the sensor the entire surface of the sensor was fixed to the heat exchanger (including electrodes). The exchanger was mounted directly on the cold finger of the cryocooler (second stage of the cryocooler) (Figure 6). 

The temperature of heat exchanger was measured and controlled by a precise Lakeshore 331 temperature controller and the reference sensors (silicon diodes DT-670-SD, Lakeshore Cryotronics Inc., Westerville, OH, USA). The glass-epoxy laminate G10 (MG Chemicals, Burlington, ON, Canada) was used for preparing the fastening and support elements. The observation of the temperature effect on resistivity of the graphene sensors was the main research work. Resistivity measurements were performed using a Keysight 34420A Micro-Ohm Meter (4-probe method, Keysight Technologies, Santa Rosa, CA, USA). The resistivity of sensors before and after encapsulation has been measured in wide range of temperature (20–295 K). This allowed observing any influence of the encapsulation process on electrical parameters of graphene. 

## 3. Results and Discussion

### 3.1. Raman Spectroscopy

**Figure 7:**
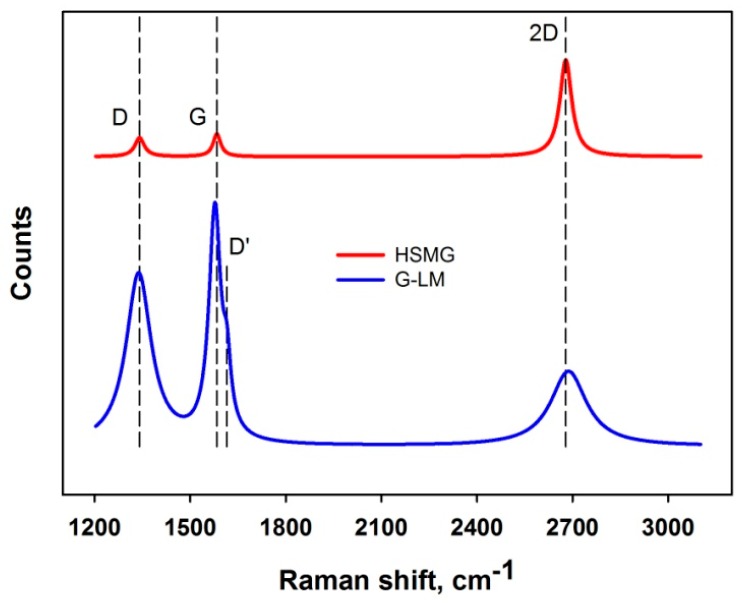
Raman spectra of single layer of high strength metallurgical graphene (HSMG^®^)—red curve and metallurgical multi-layered graphene-like material (G-LM)—blue curve.

Spectra for the studied graphene structures are presented on Figure 7.

The spectrum of HSMG^®^ graphene consists of characteristic G and 2D peaks. The intensity ratios I_G_/I_2D_ or I_2D_/I_G_ are 0.2 and 4, respectively. According to the studies of Das and Ferrari it can be noted that these values correspond to a single layer of graphene [71,72]. The additional D peak which can be observed should not appear for perfect graphene (without defects), so the presence of the D peak indicates carbon atom disorders or defects such as edges, dislocations, cracks or vacancies [73]. Taking the above into consideration, we can conclude the HSMG^®^ is defected, single-layer graphene.

The spectrum shape of G-LM graphene looks like a spectrum of graphene oxide (GO) or reduced graphene oxide (rGO) with a distinctive arrangement and shape of the G and D peaks [73,74,75]. In most papers on GO/rGO, the spectra have a completely different shape of the 2D peak, which intensity is low and the shape is blurred. Deconvolution of the 2D peak for GO allows one to extract an additional D + G peak [73,74,75,76]. It should be noted that the manufacturing process for HSMG^®^ graphene and post-processing procedures preclude the formation of graphene oxide. In G-LM graphene spectra a 2D symmetrical peak can be observed. The 2D peak for G-LM graphene is broadened in comparison to the peak for HSMG^®^ graphene. On analysis of the Raman spectrum an additional D’ peak can be also observed. The D’ peak, like the D peak, indicates a defect and a low ordered carbon structure [77]. Das in his paper shows the evolution of the Raman spectrum as the number of graphene layers increases from single layer graphene to the characteristic spectrum for graphite with a ratio I_G_/I_2D_ equal to 3.1 [72]. The mentioned value is the same in case of G-LM but it should be noted that 2D peak shape is completely different compared to graphite. For Raman spectroscopy analysis the ratio of peak intensity is very important but the shape of the 2D peak should also be taken into account. A similar Raman spectrum to that of G-LM was described by Pimenta and defined as a nanographitic structure. However, the peak 2D is narrow compared to the G-LM 2D peak and it has an intensity almost equal to the G peak intensity [77]. Taking into account the above considerations we can say that we are dealing with a disordered, multi-layer graphene-like material structure.

### 3.2. Temperature Dependence of the Resistance

**Figure 8:**
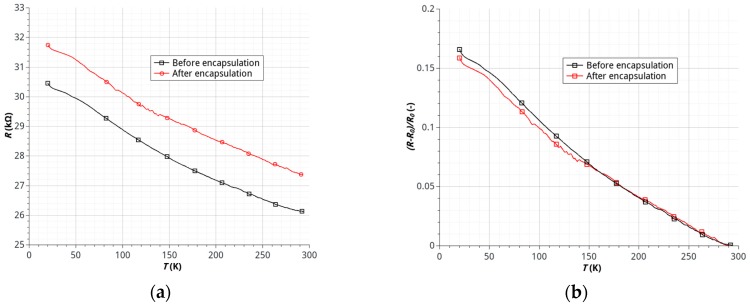
Temperature dependence of resistance of HSMG^®^ sensors before and after encapsulation: (**a**) resistance *R* (kΩ) of sensor; (**b**) relative changes of sensor resistance (*R_o_*—resistance value in 295 K).

**Figure 9:**
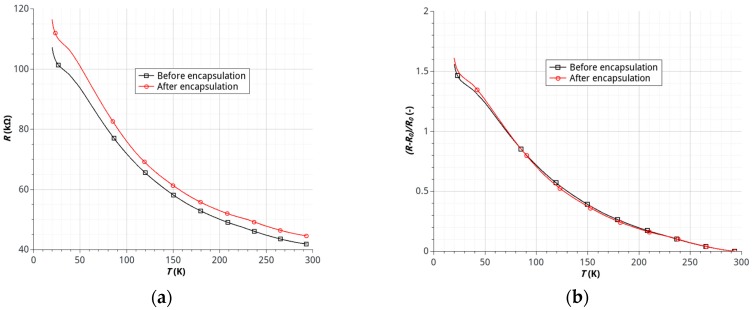
Temperature dependence of resistance of G-LM sensors before and after encapsulation: (**a**) resistance *R* (kΩ) of sensor; (**b**) relative changes of sensor resistance (*R_o_*—resistance value in 295 K).

The results of studies on the effects of temperature on the resistance of samples made of HSMG^®^ and G-LM showed a significant dependence of resistance on temperature in the range of 20–195 K characterized by a continuous, nearly linear decrease of the resistance (negative temperature coefficient of resistance). The process of encapsulation for samples with HSMG^®^ and G-LM caused the relatively small, permanent increase in the resistance of the sensor (Figure 8 and Figure 9). 

The increase in resistance of the graphene-electrode interface is the most likely cause of this effect. The connection resulting from, inter alia, the occurrence of weak intermolecular van der Waals forces may undergo a slight degradation. It should be noted, however, that a change in the resistance value after the encapsulation does not affect the nature of the temperature dependence of the resistance (Figure 8 and Figure 9). This means that the process does not degrade the active layer of the sensor. In the case of the sensor made of the HSMG^®^ layer the observed change in resistance of the sensor is on the order of 16% (Figure 8). In the case of G-LM sensors a much stronger dependence, on the order of 150%, was observed (Figure 9). The nature of the changes of resistance is less linear than for HSMG^®^ sensors. It should be noted that, for temperatures of less than 150 K, a growth rate of the resistance change was observed, which translates directly into increased sensitivity of the sensor in this temperature range.

## 4. Conclusions

The observation of the effect of temperature on the electrical characteristics of graphene indicates the potential use of graphene layers in the construction of temperature sensors. The temperature characteristics of the analyzed graphene sensors exhibit no clear anomalies or strong non-linearity in the entire studied temperature range (as compared to the typical carbon sensor). The as-fabricated sensors have a low thermal capacity due to their construction. Changes in resistance of sensors using HSMG^®^ and G-LM layers are measurable and significant. The sensors’ sensitivity in the range of cryogenic temperatures (particularly below 50 K) is much higher than the sensitivity of comparable resistive (metallic) temperature sensors (RTD). Measurements carried out at temperatures below approximately 20 K showed the increase of dynamics of the sensors’ resistance changes with temperature. It should be noted that the measured changes of electrical resistance as a temperature function in the case of the sensor based on a monolayer graphene are significantly less than for a sensor prepared using a defected graphene-like material. The explanation of this phenomenon requires further study. Temperature sensors made of the graphene film transferred on a polymeric substrate have significantly better flexibility and resistance to thermal stresses in comparison to bulk graphite sensors. The application of thin conductive polymeric films (ITO and AgHT^TM^) allowed obtaining sensors with electrodes characterized by high optical transparency. These sensors may be applicable in low-temperature optical studies (UV). In cases where transparency is not particularly required, Kapton tape with Au electrodes can be used because of its good electrical and thermal properties in cryogenic temperatures. Future studies of sensors placed in a controlled atmosphere (gas/vacuum in the capsule) are planned.

## Figures and Tables

**Table 1:** Names of Raman peaks identified in the studied graphene structures with their specific frequencies.

Peak Name	HSMG^®^	G-LM
	ω [cm^−1^]	
D	1341.1	1338.3
G	1583.8	1577.6
D’	-	1615.4
2D	2678.8	2687.3

**Table 2:** FWHM (full width at half maximum) values and ratios of typical peaks calculated on the basis of Raman spectra deconvolution.

Peak Name	HSMG^®^			G-LM		
	FWHM [cm^−1^]	I_G_/I_2D_	I_2D_/I_G_	FWHM [cm^−1^]	I_G_/I_2D_	I_2D_/I_G_
D	36	0.2	4.1	96	3.1	0.3
G	29			45		
D’	-			29		
2D	45			136		
